# Trends in Clinician Burnout With Associated Mitigating and Aggravating Factors During the COVID-19 Pandemic

**DOI:** 10.1001/jamahealthforum.2022.4163

**Published:** 2022-11-23

**Authors:** Mark Linzer, Jill O. Jin, Purva Shah, Martin Stillman, Roger Brown, Sara Poplau, Nancy Nankivil, Kyra Cappelucci, Christine A. Sinsky

**Affiliations:** 1 Department of Medicine, Hennepin Healthcare, Minneapolis, Minnesota; 2 Department of Medicine, Northwestern University Feinberg School of Medicine, Chicago, Illinois; 3 American Medical Association, Chicago, Illinois; 4 University of Wisconsin School of Nursing, Madison

## Abstract

**Importance:**

The COVID-19 pandemic has affected clinician health and retention.

**Objective:**

To describe trends in burnout from 2019 through 2021 with associated mitigating and aggravating factors.

**Design, Setting, and Participants:**

Cross-sectional surveys were sent to physicians and advanced practice clinicians throughout 120 large US health care organizations between February 2019 and December 2021. From 56 090 surveys, there were 20 627 respondents.

**Exposures:**

Work conditions and COVID-19.

**Main Outcomes and Measures:**

Surveys measured time pressure, chaos, work control, teamwork, electronic health record use, values alignment, satisfaction, burnout, intent to leave, and in 2021, feeling valued. Multivariate regressions controlling for gender, race and ethnicity, years in practice, and role determined burnout, satisfaction, and intent-to-leave correlates.

**Results:**

Of the 20 627 respondents (median response rate, 58% [IQR, 34%-86%; difference, 52%]), 67% were physicians, 51% female, and 66% White. Burnout was 45% in 2019, 40% to 45% in early 2020, 50% in late 2020, and 60% in late 2021. Intent to leave increased from 24% in 2019 to more than 40% as job satisfaction decreased. Higher burnout was seen in chaotic workplaces (odds ratio [OR], 1.51; 95% CI, 1.38-1.66; *P* < .001) and with low work control (OR, 2.10; 95% CI, 1.91-2.30; *P* < .001). Higher burnout was associated with poor teamwork (OR, 2.08; 95% CI, 1.78-2.43; *P* < .001), while feeling valued was associated with lower burnout (OR, 0.22; 95% CI, 0.18-0.27; *P* < .001). In time trends, burnout was consistently higher with chaos and poor work control. For example, in the fourth quarter of 2021 burnout was 36% (95% CI, 31%-42%) in calm environments vs 78% (95% CI, 73%-84%) if chaotic (absolute difference, 42%; 95% CI, 34%-49%; *P* < .001), and 39% (95% CI, 33%-44%) with good work control vs 75% (95% CI, 69%-81%) if poor (absolute difference, 36%; 95% CI, 27%-44%; *P* < .001). Good teamwork was associated with lower burnout rates (49%; 95% CI, 44%-54%) vs poor teamwork (88%; 95% CI, 80%-97%; absolute difference, 39%; 95% CI, 29%-48%; *P* < .001), as was feeling valued (37%; 95% CI, 31%-44%) vs not feeling valued (69%; 95% CI, 63%-74%; absolute difference, 32%; 95% CI, 22%-39%; *P* < .001).

**Conclusions and Relevance:**

Results of this survey study show that in 2020 through 2021, burnout and intent to leave gradually increased, rose sharply in late 2021, and varied by chaos, work control, teamwork, and feeling valued. Monitoring these variables could provide mechanisms for worker protection.

## Key Points

**Question:**

How have clinician burnout rates changed during the COVID-19 pandemic nationally, and what are the key aggravators and mitigators of burnout?

**Findings:**

In this survey study of US clinicians with 20 627 respondents, burnout increased throughout the pandemic, reaching its highest levels (>60%) late in 2021; intent to leave also reached high levels (>40%) late in 2021. Chaotic workplaces and lack of control of workload were associated with higher burnout, while efficient teamwork and feeling valued were associated with lower burnout.

**Meaning:**

Knowledge of key indicators of a healthy workplace, such as work control, feeling valued, and clinician outcomes (eg, burnout, satisfaction, intent to leave) may help health systems and their workers adapt to stressful times.

## Teaser

This survey study describes trends in clinician burnout from 2019 to 2021 and associated mitigating and aggravating factors.

## Introduction

The COVID-19 pandemic has produced unparalleled changes in clinician work life, with associated worsening in stress and burnout.^1,2^ Since the start of the pandemic, clinicians have faced new sources of stress, including fear of the virus, inability to modulate workload, changes in childcare and eldercare responsibilities, requirements for care that is felt to be ethically untenable (such as rationing), and some degree of questioning meaning and purpose.^2,3,4^ These stressors have led to clinician exhaustion and burnout, and a degree of turnover and exit from practice that is unsustainable.^5,6^ However, there are to date few multicenter data or large prospective data sets that provide a clear picture of changes (trends) in clinician reactions throughout the pandemic, nor any recent data on work conditions associated with clinician coping with the stress of 2 years of pandemic work life.

In this survey study, a national data set was used to determine (1) trends before and during the pandemic in burnout, satisfaction, and intent to leave the job, as well as (2) remediable aggravators and mitigators of these variables. These data were used to construct a set of key performance indicators (KPIs) that could be useful for organizations to protect their workforces when future surges in stress occur.

## Methods

### Sample

Between February 2019 and December 2021, 20 627 physicians, residents, and advanced practice clinicians, including nurse practitioners and physician assistants, replied to a survey sent to 56 090 individuals using the Mini Z work-life measure^7^ in more than 120 American Medical Association (AMA) partner health care systems with rolling enrollment throughout the 3-year period. These organizations distributed the survey as part of their engagement with the AMA in organizational well-being efforts. There were 204 surveys administered across 120 organizations, with no cost for participation. Individuals may have responded on more than 1 occasion if their organization was surveyed again. Gender identity was self-defined by respondents, and options included female, male, nonbinary, and those preferring not to identify gender. Race and ethnicity were self-defined by respondents as Asian or Pacific Islander (unable to separate out Pacific Islander due to it being grouped together with Asian), Hispanic or Latino (also referred to as Latinx), Native American or American Indian, non-Hispanic Black (hereafter, Black), non-Hispanic White (hereafter White), other race or ethnicity (not specified by individuals), or those preferring not to identify race or ethnicity. Because data were collected on gender, race, and ethnicity, we were able to control for these variables in multivariate regressions assessing correlates of clinician outcomes. Survey distribution generally followed American Association for Public Opinion Research guidelines, with areas of discordance noted in the Limitations section. Data were deidentified and, therefore, no institutional review board approval was required, which was also confirmed by the institutional review board at Hennepin Healthcare. The project is an ongoing series of program evaluations. Each organization completes a data-use agreement allowing data to be aggregated for quality improvement and research, and each respondent receives a statement on use of the data and can opt-out of the survey at that time.

### Study Design

The convenience sample included all surveys of those who responded. Most of these US organizations (academic, community based, Federally Qualified Health Centers, and integrated delivery systems) had more than 100 clinicians. Organizations performed their own surveys, and results were pooled at the AMA-associated data lab, Forward Health Group in Madison, Wisconsin.

### Measure

The Mini Z measure is used throughout the world and recently had validation parameters published in the *Journal of General Internal Medicine*.^7^ The 10-item measure draws on constructs from the Physician Worklife Study,^8^ the MEMO (Minimizing Error, Maximizing Outcome) study,^6^ and the Healthy Work Place trial,^9^ including 3 clinician outcomes (satisfaction, stress, and burnout) and 7 known predictors (values alignment, teamwork, work control, time pressure, work pace [chaos], and factors associated with the electronic health record [EHR]). Burnout is measured with a single 5-choice question^8^ asking respondents to use their own definition of burnout. The burnout item has been validated against emotional exhaustion in the Maslach Burnout Inventory.^10^ Choices 1, 2, and 3 all mention burnout and, as is the convention,^7,11^ were used to indicate burnout. In validation studies, Cronbach α for the bifactor Mini Z model was excellent, and factor analysis showed good fit indices^7^ with validity confirmed with correlations of most work conditions with the Patient Health Questionnaire-2 depression measure and of the respondent’s sense of documentation time pressure and excess home EHR use with 2 measures of self-noted EHR use (EHR work outside of work and hours per week of indirect patient care including EHR-related work).

During the first year of the pandemic (April 2020-April 2021), a Coping with COVID survey was modeled after the Mini Z to capture work-life factors associated with pandemic-related stress.^1,2,3^ Internal consistency was good, as was construct validity.^2,3^ In the middle of the pandemic, a question on intent to leave the organization^8^ was added (answers of moderately likely, likely, or definite indicated an intent to leave). Several questions from the Coping with COVID survey were added later in the pandemic; the one included in this report (based on a sufficient sample of respondents and important prior findings^1,2,3,4^) was sense of feeling valued by one’s organization. Because these questions (intent to leave and feeling valued) were added over time, the number responding to these questions varied. Aside from these 2 questions, missing data were minimal. As data were not missing at random, no imputations were performed. Detailed findings from the Coping with COVID survey have been previously reported.^1,2,3,4^

### Statistical Analysis

Data are dated from when respondents completed and returned their surveys. Trends in burnout were assessed using linear longitudinal analysis of data, with changes in burnout trends assessed visually and statistically for potential aggravators and mitigators selected from prior studies, focusing on work conditions such as fast-paced, chaotic workplaces and lack of work control^6^ (aggravators), and teamwork and feeling valued^2,3,4^ (mitigators). Other variables included values alignment with leaders^6^ (mitigator) and challenges with EHRs^11^ (aggravator). Trends in burnout by aggravators and mitigators, adjusted for gender, race and ethnicity, years in practice, and role, were represented visually using polynomial splines as well as linear functions. To assess the role of aggravators and mitigators, differences in clinician outcome linear trend lines over time were assessed via *t* tests to compare slopes and intercepts of best-fit lines. No extrapolation was performed. Potentially influential work conditions included chaos, control, teamwork, EHR use, values alignment, and feeling valued, and were assessed via 2-level (individuals nested within organizations) logistic regressions for associations with clinician outcomes. Models were conducted separately for feeling valued given a smaller sample of respondents. Significance was assessed for a 2-tailed α at *P* < .05. Analyses were conducted using NCSS statistical software (2019) and Stata, version 17.0 (StataCorp).

## Results

### Sample

From 56 090 surveys, there were 20 627 respondents (average response rate, 37%; median response rate across organizations, 58% [IQR, 34%-86%; difference, 52%]), with 67% of respondents being physicians, 51% female, 66% White, and 38% practicing 10 years or fewer (Table 1). Respondents were widely located, with 20% (n = 4026) in the Northeast, 9% (n = 1887) in the Midwest, 29% (n = 6034) in the South, 24% (n = 5045) in the West, and 18% (n = 3618) missing.

**Table 1.** Demographics of Mini Z–Surveyed Populations, 2019-2021 (N = 20 627)

Characteristic	No. (%)
Gender^a^	
Female	10 576 (51.3)
Male	8157 (39.5)
Nonbinary/third gender	36 (0.2)
Prefer not to answer	852 (4.1)
Missing	1006 (4.9)
Race and ethnicity^a^	
Asian or Pacific Islander	1774 (8.6)
Black	627 (3.0)
Hispanic or Latinx	718 (3.5)
Native American or American Indian	45 (0.2)
White	13 685 (66.3)
Other (not specified)	331 (1.6)
Prefer not to answer	1293 (6.3)
Missing	2154 (10.4)
Role	
Physician	13 736 (66.6)
Nurse practitioner/physician assistant	4800 (23.3)
Other advance practice clinician	1328 (6.4)
Missing	763 (3.7)
Years in practice since training, y	
1-5	4502 (21.8)
6-10	3368 (16.3)
11-15	2487 (12.1)
16-20	1954 (9.5)
>20	4815 (23.3)
NA	123 (0.6)
Missing	3378 (16.4)
Medical specialty	
Allergy and immunology	27 (0.1)
Anesthesiology	846 (4.1)
Cardiac/thoracic surgery	108 (0.5)
Cardiovascular diseases	522 (2.5)
Critical care medicine	231 (1.1)
Dentistry/oral surgery	128 (0.6)
Dermatology	102 (0.5)
Emergency medicine	1116 (5.4)
Family medicine	3752 (18.2)
Gastroenterology	335 (1.6)
General practice	144 (0.7)
Hematology/oncology	467 (2.3)
Hospitalist	949 (4.6)
Infectious disease	150 (0.7)
Internal medicine, general/primary care	1690 (8.2)
Nephrology	168 (0.8)
Neurological surgery	114 (0.6)
Neurology	323 (1.6)
Obstetrics and gynecology	1410 (6.8)
Oncology	172 (0.8)
Ophthalmology	144 (0.7)
Orthopedic surgery	454 (2.2)
Otolaryngology	167 (0.8)
Palliative care	160 (0.8)
Pathology	194 (0.9)
Pediatrics	1579 (7.7)
Physical and occupational therapy	18 (0.1)
Physical medicine and rehabilitation	135 (0.7)
Plastic surgery	51 (0.2)
Podiatry	102 (0.5)
Psychiatry	510 (2.5)
Pulmonary disease	200 (1.0)
Radiation oncology	75 (0.4)
Radiology	409 (2.0)
Rheumatology	86 (0.4)
Surgery, general	497 (2.4)
Urological surgery	182 (0.9)
Vascular surgery	66 (0.3)
Other surgery-related specialty	217 (1.1)
Other nonsurgery-related specialty	667 (3.2)
NA	381 (1.8)
Missing	1579 (7.7)

Abbreviation: NA, not available.

^a^ Self-defined by respondents.

### Burnout Rates

**Figure 1.**
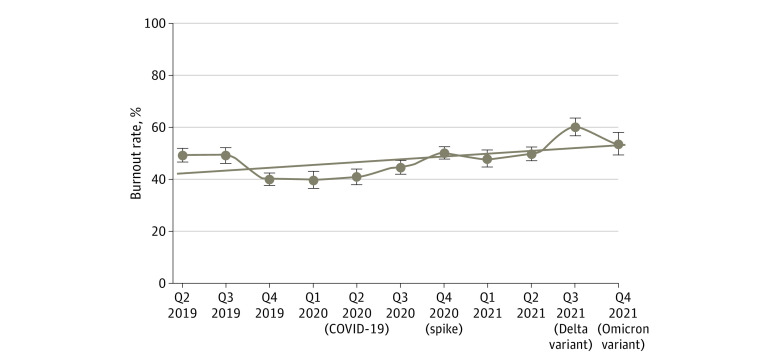
Trends in Clinician Burnout, 2019-2021 Lines represent simple linear functions to the data, dots represent the average proportional values per time period, and whiskers represent 95% CIs around the average proportional values.

Overall burnout rate across the 3-year time period was 49%. Burnout was 45% in 2019 and declined through the year. There was a brief rise in early 2020 followed by a decrease during the early phases of the pandemic (40%-45%). Burnout increased toward the end of 2020 (50%), generally worsened throughout 2021, and showed a steep rise to the highest levels ever recorded, to our knowledge, by the fourth quarter of 2021 (approximately 60%). Compared with prepandemic levels, there was a statistically significant increase in burnout in 2020 through 2021 (51% vs 45%; odds ratio [OR], 1.27; 95% CI, 1.20-1.35; *P* < .001). Figure 1 and eTable 1 in the Supplement show longitudinal trends in burnout before and throughout the pandemic. Trends in satisfaction (eFigure 1 in the Supplement) and intent to leave (eFigure 2 in the Supplement) showed drops in satisfaction and elevations in intent to leave toward the end of 2021.

### Time Trends in Burnout With Aggravators and Mitigators

**Figure 2.**
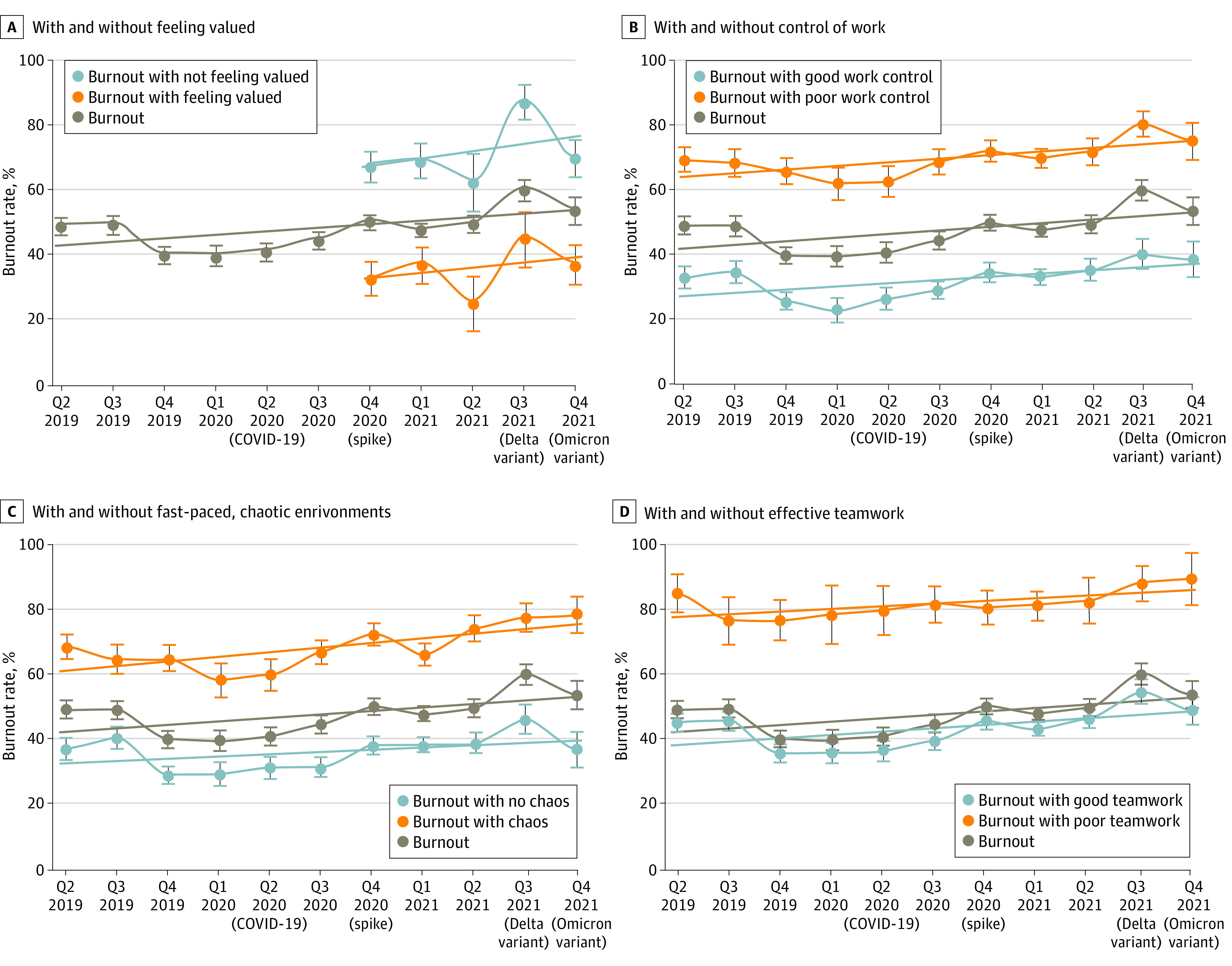
Variation in Burnout Trends by Key Aggravators and Mitigators Lines represent simple linear functions to the data, dots represent the average proportional values per time period, and whiskers represent 95% CIs around the average proportional values.

Although burnout rates increased, they did so at lower levels if mitigators such as teamwork or feeling valued were present. Figure 2 demonstrates trends in burnout over time occurring at lower burnout levels in those feeling valued vs those not feeling valued: in the fourth quarter of 2021, burnout was 37% (95% CI, 31%-43%) when feeling valued vs 69% (95% CI, 63%-74%) when not (absolute difference, 32%; 95% CI, 22%-39%; *P* < .001). Similarly, burnout occurred at higher levels in those lacking work control vs those describing good control: 75% (95% CI, 69%-81%) vs 39% (95% CI, 33%-44%), respectively (absolute difference, 36%; 95% CI, 27%-44%; *P* < .001). Figure 2C and D show similar findings for chaos and teamwork, with lower burnout in calmer environments vs chaotic environments (36% [95% CI, 31%-42%] vs 78% [95% CI, 73%-84%]; absolute difference, 42% [95% CI, 34%-49%]; *P* < .001) and for those experiencing good teamwork vs poor teamwork (49% [95% CI, 44%-54%] vs 88% [95% CI, 80%-97%]; absolute difference, 39% [95% CI, 29%-48%]; *P* < .001). Of note, teamwork burnout curves (Figure 2D) show modest improvement with good teamwork but substantially higher burnout when teamwork is poor. eFigure 3 in the Supplement shows comparable findings for values alignment (mitigator) and excessive home EHR use (aggravator). eTable 2 in the Supplement summarizes differences in intercepts and slopes for aggravators and mitigators; slopes are for the most part comparable, demonstrating that rates of burnout increased in all groups (those with and without aggravators or mitigators).

### Time Trends in Satisfaction and Intent to Leave With Aggravators and Mitigators

There were comparable trend shifts for satisfaction with feeling valued, work control, and excessive home EHR use (eFigure 4 in the Supplement), and for intent to leave with feeling valued, control of workload, and good teamwork (eFigure 5 in the Supplement). There were substantive differences in levels of satisfaction and intent to leave over time, with high adverse outcomes (intent to leave) with aggravators and improved outcomes with mitigators.

### Comparison of Primary vs Subspecialty Care

Trends in burnout for primary care specialties vs subspecialty care (eFigure 6 in the Supplement) show evidence for increasing rates in both over time. Those in primary care experienced higher burnout rates than subspecialists earlier on, but the difference narrowed over time.

### Multivariate Regressions of Aggravating and Mitigating Factors Associated With Burnout

In regression analyses (Table 2), several variables correlated with higher burnout, with 45% of variance in burnout explained by the model. The McKelvey and Zavoina pseudo-*R*^2^ assumes the existence of an underlying continuous dependent variable that, if measured, would satisfy a linear model; subsequently, the pseudo-*R*^2^ may be interpreted similarly to an ordinary least squares regression *R*^2^ and approximates the percentage variance in outcomes explained by the models. Aggravators included high chaos (OR, 1.51; 95% CI, 1.38-1.66; *P* < .001), lack of work control (OR, 2.10; 95% CI, 1.91-2.30; *P* = .001), and excessive home EHR use (OR, 1.30; 95% CI, 1.17-1.44; *P* < .001). Mitigators included feeling valued (OR, 0.22; 95% CI, 0.18-0.27; *P* < .001), good teamwork, and values alignment with leaders. Controlling for geographic region made no appreciable difference in these findings. Table 2 summarizes adjusted relative risks, which, given the high prevalence of burnout (50%), are lower than ORs. Even using adjusted relative risks, the associated absolute increments in burnout were generally more than 5%, which was felt to be a clinically meaningful difference.

**Table 2.** Multivariable Regressions Assessing Factors Associated With Burnout

Factor	AOR (95% CI)	*P* value	Organizational surveys (n = 134)^a^	
			ARR (95% CI)	ARD (95% CI)
Poor values alignment	2.78 (2.42-3.20)	<.001	1.37 (1.32-1.43)	0.16 (0.14-0.19)
Poor teamwork	2.08 (1.78-2.43)	<.001	1.25 (1.20-1.31)	0.11 (0.09-0.14)
High stress^b^	6.36 (5.84-6.93)	<.001	2.25 (2.15-2.35)	0.36 (0.34-0.38)
Excessive home EHR use	1.30 (1.17-1.44)	<.001	1.09 (1.05-1.13)	0.04 (0.02-0.05)
High documentation time pressure	1.47 (1.32-1.63)	<.001	1.14 (1.10-1.18)	0.06 (0.04-0.07)
High level of chaos	1.51 (1.38-1.66)	<.001	1.15 (1.11-1.18)	0.06 (0.05-0.08)
Poor work control	2.10 (1.91-2.30)	<.001	1.29 (1.25-1.34)	0.12 (0.10-0.14)
Random effects, organization variable^c^	0.065 (0.032-0.131)	NA	NA	NA

Abbreviations: AOR, adjusted odds ratio; ARD, adjusted risk difference; ARR, adjusted relative risk; EHR, electronic health record; NA, not applicable.

^a^ A total of 14 397 observations were recorded.

^b^ The ARR estimate on the variable stress can be interpreted as those individuals with high stress are 2.25 times more likely to be at risk for burnout, holding other variables constant. The ARD, an absolute risk measure, can be interpreted as stressed individuals having 36 percentage points greater burnout than nonstressed individuals. Because burnout was frequent in the study sample, the AOR of 6.36 was further from 1.0 than the ARR of 2.25.

^c^ McKelvey and Zavoina pseudo-*R*^2^ = 0.45.

Forty-two percent of variance in satisfaction was explained by the regression model (eTable 3 in the Supplement), with work control, teamwork, and values alignment correlating with satisfaction. The feeling-valued model showed a strong correlation with improved satisfaction (OR, 10.33; 95% CI, 8.02-13.30; *P* < .001; pseudo-*R*^2^, 34%). A third logistic regression model explained 23% of variance in intent to leave (eTable 4 in the Supplement) with associated variables including teamwork, values alignment, and work control. Feeling-valued models again demonstrated meaningful correlations (OR, 0.21; 95%CI, 0.16-0.25; *P* < .001); however, only 45% of clinicians in 2021 felt valued by their organizations.

## Discussion

In this survey study of more than 20 000 clinician responses before and during the COVID-19 pandemic, levels of burnout were high and fairly stable during early 2020, with slight rises late in 2020 and a sharp rise late in 2021; likewise, intent to leave was stable at moderate levels in 2020 and 2021 until a steep rise toward the end of 2021. The lack of increase in burnout through the difficult year of 2020 is notable and may indicate a sense of determination and purpose among these professionals. However, the data show how the persistent lack of control of workload, chaotic environments, challenges with teamwork, and a lack of feeling valued by organizations may have contributed to worsening burnout and a rise in intent to leave. Being knowledgeable of such trends in burnout, seen also in non–health care workers during this challenging time period, and the associated work-life characteristics could allow organizations to make adjustments in real time to modulate workload, reduce chaos, and improve teamwork and sense of value, thus providing a meaningful opportunity to address burnout and retention.

### Trends in Burnout, Satisfaction, and Intent to Leave

After some degree of national attention,^12^ burnout rates prior to the pandemic were falling in late 2019 and early 2020. At the start of the pandemic, there was a small, further decrease in burnout and intent to leave as health care workers came together for a common goal. As time wore on, however, rates of burnout and intent to leave rose,^13,14^ and in late 2021, there were unprecedented levels of burnout (>60%) and intent to leave the job (>40%), perhaps due to a combination of depletion, exhaustion, and the sense of no longer being valued by organizations or even patients. This late 2021 peak in burnout occurred concurrently with a national rise in work departures known as the Great Resignation. Preventing such occurrences going forward may be possible as we gain greater knowledge of aggravators and mitigators of burnout.

### Aggravators and Mitigators of Burnout, Satisfaction, and Intent to Leave

Variables related to higher levels of burnout and intent to leave included chaotic (fast-paced) workplaces and lack of control of workload. Absolute differences in burnout between favorable and unfavorable environments were 30% or higher, which is notable. Understanding and managing work pace^9^ and modulating workload by giving clinicians greater control of their own schedules^15^ could mitigate stress seen during COVID-19 surges.

Meanwhile, feeling valued and good teamwork were associated with favorable outcomes. How to make clinicians feel valued is being actively explored,^16^ but general principles include having a receptive leadership team who listens to frontline workers and makes tangible changes based on feedback and needs, and providing organizational support for work-life integration, as well as clinician self-care. The benefits could be substantial, as only 45% of clinicians felt valued in 2021. Good teamwork refers to not only team member camaraderie and a positive team culture, but also solid team-based care workflows that allow for efficient task-sharing and minimizing non–patient-facing tasks for clinicians.

### Policy Implications

To protect the health care workforce, we propose synthesizing a set of KPIs; these could include work stressors (eg, chaos, lack of work control), burnout mitigators (eg, teamwork, feeling valued), and clinician outcomes (eg, burnout, satisfaction, intent to leave). Satisfaction, a proxy for joy in work, is included because it is known to be connected with intent to leave a job.^17^ Other variables indicative of a healthy workplace could include making more explicit values alignment with leaders and minimizing stress from the EHR.^11^ With recommendations to put burnout on organizational dashboards,^18,19^ the KPI strategy could provide measurable and remediable work conditions and outcomes to guide optimizing a work environment for protection of the health care team. One new aspect is that the dashboard should not overly focus on the percentage of burned-out health care workers, but rather the remediable aggravators and mitigators of burnout (eg, chaotic working conditions, work control, feeling valued). Because of the pandemic’s persistence, caring for health care professionals and other staff is more important than ever. The KPI measures could serve as an early warning system during times of stress to identify challenges and opportunities to improve the work environment. Given annual cost estimates of approximately $5 billion for burnout-related US physician turnover,^5,20^ cost savings could be considerable. Clinicians may work more and burn out because they perceive no options to work less, suggesting it is the structure of US health care that, in part, limits the ability to mitigate burnout. This study offers data to support targeted processes to bring sense to a health care system that wishes to preserve and sustain its workers.

### Strengths and Limitations

While response rates were generally favorable (58% is high for a national clinician study^12^), there are several limitations to this study, including the use of a convenience sample, the inability to determine aspects of nonrespondents or of the organizations themselves, surveys administered sporadically at the discretion of enrolling organizations, and deployment of Coping with COVID questions only later in the pandemic. Because data are predominantly cross-sectional, causality cannot be inferred; however, the strength of associations, and the longitudinal data collection with durable increments in burnout in association with work-life variables, supports the meaningfulness of these variables in relation to clinician outcomes. Finally, while the single-item burnout measure is associated mainly with emotional exhaustion, the other items of the Mini Z correlate well with depersonalization on the Maslach Burnout Inventory.^21^

## Conclusions

Results of this survey study suggest that a federal surveillance system with real-time analysis of levels of clinician and health care worker outcomes could be very meaningful in addressing the current state of burnout, dissatisfaction, and potential job loss.^22^ Despite an epidemic of burnout prior to the pandemic^23^ and clear recognition during the pandemic of the toll on health care workers,^2^ many organizations may not be aware of effective ways to approach burnout reduction. We hope that these data will allow a national conversation, along the lines begun by the National Academy of Medicine^24^ and the Office of the US Surgeon General,^25,26^ to highlight, measure, and correct work conditions to improve clinician stress, satisfaction, morale, retention, and ultimately, the quality of patient care.

## Notes

Supplement. **eTable 1.** Burnout frequency time counts **eTable 2.** Best fit line intercepts and slopes for aggravators and mitigators **eTable 3.** Multivariate regressions assessing factors related to satisfaction **eTable 4.** Multivariate regressions assessing factors related to intent to leave **eFigure 1.** Trends in satisfaction **eFigure 2.** Trends in intent to leave **eFigure 3.** Trends in burnout with high vs low values alignment with leaders and excessive home electronic health record (EHR) use **eFigure 4.** Trends in satisfaction with feeling valued and changes in satisfaction with high vs low work control and excessive home electronic health record (EHR) use **eFigure 5.** Trends in intent to leave with feeling valued vs not feeling valued and changes in intent to leave with high vs low work control and good vs poor teamwork **eFigure 6.** Primary care vs subspecialty care trends in burnout 2019-21
