# Error in Abstract

**DOI:** 10.1001/jamahealthforum.2023.0002

**Published:** 2023-02-10

In the Original Investigation titled “Trends in Clinician Burnout With Associated Mitigating and Aggravating Factors During the COVID-19 Pandemic,”^1^ published November 23, 2022, it was incorrectly stated in the Results section of the Abstract that 24% of physician respondents expressed an intent to leave their organization in 2019. The correct percentage is 30%. This article has been corrected.

## References

[1] Linzer M, Jin JO, Shah P, . Trends in clinician burnout with associated mitigating and aggravating factors during the COVID-19 pandemic. JAMA Health Forum. 2022;3(11):e224163. doi:10.1001/jamahealthforum.2022.4163 36416816 PMC9685483

