# Supplementary material for: Trends in Clinician Burnout With Associated Mitigating and Aggravating Factors During the COVID-19 Pandemic
Source: JAMA Health Forum. 2022 Nov 23;3(11):e224163. doi: 10.1001/jamahealthforum.2022.4163 (PMC9685483; doi:10.1001/jamahealthforum.2022.4163)
Supplement: Supplement. — eTable 1. Burnout frequency time counts eTable 2. Best fit line intercepts and slopes for aggravators and mitigators eTable 3. Multivariate regressions assessing factors related to satisfaction eTable 4. Multivariate regressions assessing factors related to intent to leave eFigure 1. Trends in satisfaction eFigure 2. Trends in intent to leave eFigure 3. Trends in burnout with high vs low values alignment with leaders and excessive home electronic health record (EHR) use eFigure 4. Trends in satisfaction with feeling valued and changes in satisfaction with high vs low work control and excessive home electronic health record (EHR) use eFigure 5. Trends in intent to leave with feeling valued vs not feeling valued and changes in intent to leave with high vs low work control and good vs poor teamwork eFigure 6. Primary care vs subspecialty care trends in burnout 2019-21 [file jamahealthforum-e224163-s001.pdf]

Supplemental Online Content

Linzer M, Jin JO, Shah P, et al. Trends in clinician burnout with associated mitigating and aggravating factors during the COVID-19 pandemic. *JAMA Health Forum*. 2022;3(11):e224163. doi:10.1001/jamahealthforum.2022.4163

eTable 1. Burnout frequency time counts

eTable 2. Best fit line intercepts and slopes for aggravators and mitigators

eTable 3. Multivariate regressions assessing factors related to satisfaction

eTable 4. Multivariate regressions assessing factors related to intent to leave

eFigure 1. Trends in satisfaction

eFigure 2. Trends in intent to leave

eFigure 3. Trends in burnout with high vs low values alignment with leaders and excessive home electronic health record (EHR) use

eFigure 4. Trends in satisfaction with feeling valued and changes in satisfaction with high vs low work control and excessive home electronic health record (EHR) use

eFigure 5. Trends in intent to leave with feeling valued vs not feeling valued and changes in intent to leave with high vs low work control and good vs poor teamwork

eFigure 6. Primary care vs subspecialty care trends in burnout 2019-21

This supplemental material has been provided by the authors to give readers additional information about their work.

© 2022 Linzer M et al. *JAMA Health Forum.*

eTable 1. Burnout frequency time counts (turning points in bold)		
**Time**	**N**	**Mean**
Feb-19	45	0.6
Mar-19	230	0.4347826
Apr-19	1125	0.504
May-19	1224	0.4158497
Jun-19	163	0.5337423
Jul-19	308	0.512987
Aug-19	349	0.4842407
Sep-19	479	0.48643
Oct-19	221	0.3303168
Nov-19	809	0.433869
**Dec-19**	**586**	**0.3941979 (lower rate end of 2019)**
Jan-20	638	0.4169279
Feb-20	326	0.4386503
Mar-20	57	0.368421
Apr-20	26	0.5
May-20	6	0.6666667
Jun-20	1042	0.4097889
Jul-20	398	0.4773869
Aug-20	1242	0.4162641
Sep-20	584	0.5702055
**Oct-20**	**1224**	**0.5179738 (elevation early in the pandemic)**
Nov-20	814	0.485258
Dec-20	412	0.5024272
Jan-21	1051	0.4624167
Feb-21	1206	0.4933665
Mar-21	558	0.4551971
Apr-21	987	0.4812563
May-21	289	0.4844291
Jun-21	268	0.5671642
Jul-21	750	0.5093333
**Aug-21**	**298**	**0.6073825 (sudden rise late 2021)**
Sep-21	463	0.6155508
**Oct-21**	**894**	**0.6644295 (peak elevation 66%, late 2021)**
Nov-21	1117	0.5666965
**Dec-21**	**438**	**0.5410959 (suggestions of a decline toward prior levels)**
Total	20627	0.4881466 (average **burnout rate 2019-21 = 49%)**

© 2022 Linzer M et al. *JAMA Health Forum.*

eTable 2. Best fit line intercepts and slopes for aggravators and mitigators

	Burnout		Satisfaction		Intent to Leave	
	Intercept Percent Scale	Slope Percent change per quarter	Intercept Percent Scale	Slope Percent change per quarter	Intercept Percent Scale	Slope Percent change per quarter
Chaos						
Calm	31.6	0.508	85.9	-0.492	25.7	0.504
Hectic	61.1	0.512	63.2	-0.494	42.2	0.505
Diff	29.5	0.004	-22.7	-0.002	16.5	0.001
Diff 95% CI	[25.8, 33.2]	[-0.02, 0.03]	[-25.7, -19.6]	[-0.02, 0.02]	[12.9, 20.0]	[-0.02, 0.02]
p-value	<0.001	0.72	<0.001	0.87	<0.001	0.92
Teamwork						
Optimal	37.7	.508	83.6	-0.492	27.4	0.504
Poor	76.9	.513	31.0	-0.499	65.1	0.504
Diff	39.1	.47	-52.6	-0.006	37.7	0.0002
Diff 95% CI	[33.9, 44.3]	[-0.04,0.05]	[-57.8, -47.3]	[-0.04, 0.03]	[32.1, 43.2]	[-0.04, 0.04]
p-value	<0.001	0.84	<0.001	0.74	<0.001	0.99
Values Alignment						
Good	36.1	0.509	85.0	-0.492	26.0	0.503
Poor	77.2	0.510	35.3	-0.494	65.2	0.508
Diff	41.1	0.001	-49.7	-0.001	39.2	0.005
Diff 95% CI	[36.5, 45.7]	[-0.03, 0.04]	[-54.4, -44.9]	[-0.03, 0.03]	[34.4, 43.9]	[-0.03, 0.04]
p-value	<0.001	0.93	<0.001	0.91	<0.001	0.78
Stress						
Low	19.0	0.505	90.2	-0.497	21.9	0.502
High	67.9	0.511	63.9	-0.493	42.5	0.504
Diff	48.9	0.005	-26.3	0.004	20.6	0.002
Diff 95% CI	[45.7, 52.1]	[-0.02, 0.03]	[-29.0, -23.5]	[-0.02, 0.03]	[17.3, 23.8]	[-0.02, 0.03]
p-value	<0.001	0.69	<0.001	0.79	<0.001	0.84
EMR Use						
Satisfactory	29.0	0.509	83.9	-0.495	27.7	0.501
Excessive	55.7	0.512	71.7	-0.489	35.4	0.508
Diff	26.7	0.003	-12.2	0.005	7.7	0.007
Diff 95% CI	[23.2, 30.1]	[-0.02, 0.02]	[-14.9, -9.4]	[-0.02, 0.03]	[4.5, 10.8]	[-0.01, 0.03]
p-value	<0.001	0.78	<0.001	0.65	0.002	0.59
Documentation						
Good	26.3	0.514	86.0	-0.493	25.5	0.503
Poor	57.0	0.511	70.4	-0.489	36.9	0.508
Diff	30.7	-0.003	-15.6	0.004	11.4	0.005
Diff 95% CI	[27.3, 34.1]	[-0.02, 0.02]	[-18.2, -12.9]	[-0.02, 0.03]	[8.2, 14.5]	[-0.02, 0.03]
p-value	<0.001	0.78	<0.001	0.62	<0.001	0.70

© 2022 Linzer M et al. *JAMA Health Forum.*

Work Control						
Optimal	26.9	0.510	88.8	-0.496	21.7	0.504
Poor	64.0	0.511	62.1	-0.490	45.6	0.505
Diff	37.0	0.001	-26.7	0.005	23.9	0.0004
Diff 95% CI	[33.5, 40.5]	[-0.02, 0.02]	[-29.5, -23.8]	[-0.02, 0.03]	[20.5, 27.2]	[-0.02, 0.02]
p-value	<0.001	0.98	<0.001	0.71	< 0.001	0.97
Feeling Valued						
Not feeling valued	67.2	0.520	57.9	-0.471	48.4	0.534
Feeling valued	32.8	0.511	92.5	-0.470	19.7	0.507
Diff	-34.6	-0.009	38.2	0.003	-33.3	-0.02
Diff 95% CI	[--38.5, -30.8]	[-0.13, 0.11]	[35.0, 41.5]	[-0.13, 0.14]	[-36.9, -29.6]	[-0.15, 0.10)
p-value	<0.001	0.87	<0.001	0.96	<0.001	0.67

© 2022 Linzer M et al. *JAMA Health Forum.*

eTable 3. Multivariate regressions assessing factors related to satisfaction

			Number of obs = 14,397	
			Number of organizational surveys = 134	
	AOR [95% CI]	p-value	ARR [95% CI]	ARD [95% CI]
Values alignment				
Poor	0.20 [0.17, 0.22]	<0.001	0.66 [0.64, 0.69]	-0.26 [-0.28, -0.24]
Teamwork				
Poor	0.24 [0.21, 0.27]	<0.001	0.71 [0.68, 0.74]	-0.22 [-0.24, -0.19]
Stress				
stressful	0.34 [0.31, 0.38]	<0.001	0.83 [0.81, 0.85]	-0.13 [-0.15, -0.12]
Electronic Health Record use				
Excessive	0.83 [0.74, 0.93]	0.002	0.97 [0.95, 0.98]	-0.02 [-0.03, -0.007]
Documentation				
Poor	0.84 [0.74, 0.94]	0.004	0.97 [0.95, 0.99]	-0.02 [-0.03, -0.007]
Chaos level				
Chaotic	0.81 [0.73, 0.89]	<0.001	0.96 [0.94, 0.98]	-0.02 [-0.03, -0.01]
Work control				
Poor	0.39 [0.35, 0.43]	<0.001	0.84 [0.82, 0.86]	-0.12 [-0.14, -0.11]
Random Effects				
Organization Variable	0.140 [0.07, 0.24]			
McKelvey&Zavoina-Pseudo-R2 = 0.42				

© 2022 Linzer M et al. *JAMA Health Forum.*

eTable 4. Multivariate regressions assessing factors related to intent to leave

			Number of obs = 13,777	
			Number of organizational surveys = 133	
	AOR [95% CI]	p-value	ARR [95% CI]	ARD [95% CI]
Values alignment				
Poor	3.60 [3.21, 4.04]	<0.001	1.91 [1.82, 2.01]	0.27 [0.24, 0.29]
Teamwork				
Poor	2.24 [1.97, 2.54]	<0.001	1.51 [1.42, 1.60]	0.16 [0.13, 0.19]
Stress				
stressful	1.70 [1.55, 1.85]	<0.001	1.35 [1.28, 1.42]	0.10 [0.08, 0.11]
Electronic Health Record use				
Excessive	1.06 [0.96, 1.17]	0.24	1.03 [0.97, 1.09]	0.01 [-0.007, 0.02]
Documentation				
Poor	1.08 [0.97, 1.19]	0.13	1.04 [0.98, 1.10]	0.01 [-0.004, 0.03]
Chaos level				
Chaotic	1.07 [0.98, 1.17]	0.13	1.03 [0.98, 1.09]	0.02 [-0.004, 0.02]
Work control				
Poor	1.83 [1.67, 2.00]	<0.001	1.40 [1.33, 1.48]	0.11 [0.09, 0.13]
Random Effects				
Organization Variable	0.08 [0.03, 0.16]			
McKelvey&Zavoina-Pseudo-R2 = 0.23				

© 2022 Linzer M et al. *JAMA Health Forum.*

eFigure 1. Trends in satisfaction

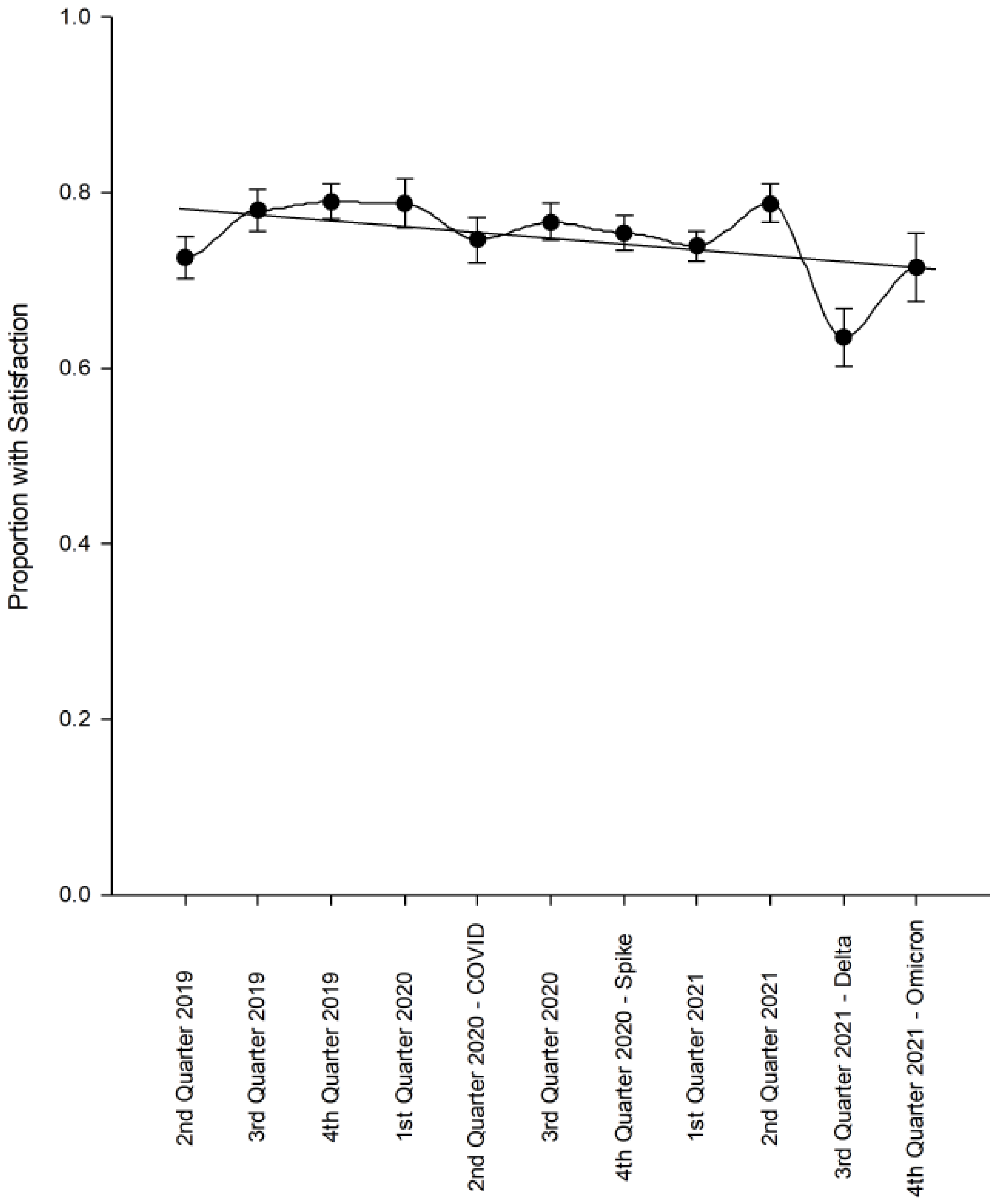

© 2022 Linzer M et al. *JAMA Health Forum.*

eFigure 2. Trends in intent to leave

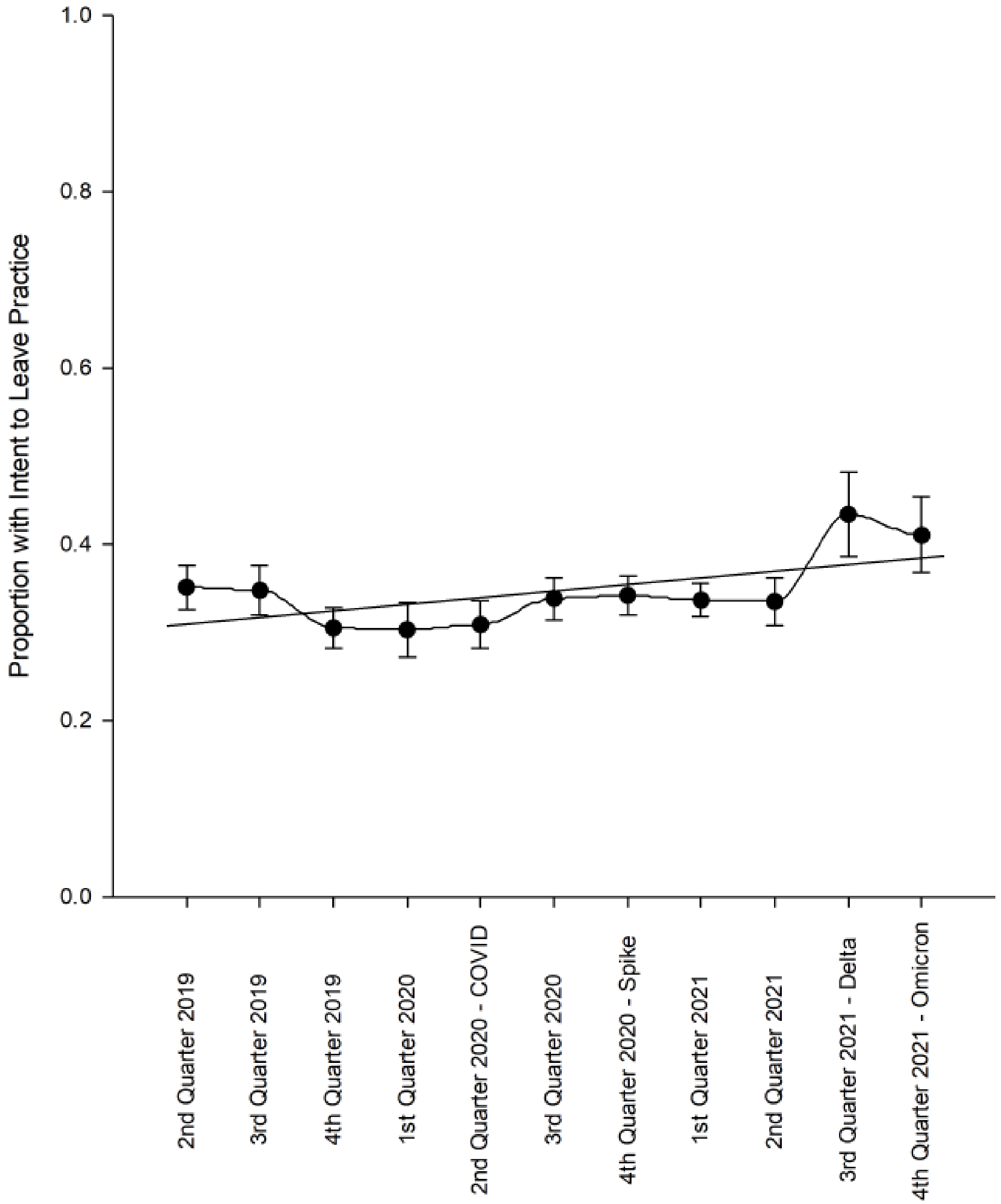

© 2022 Linzer M et al. *JAMA Health Forum.*

eFigure 3, Panel A. Trends in burnout with high vs low values alignment with leaders

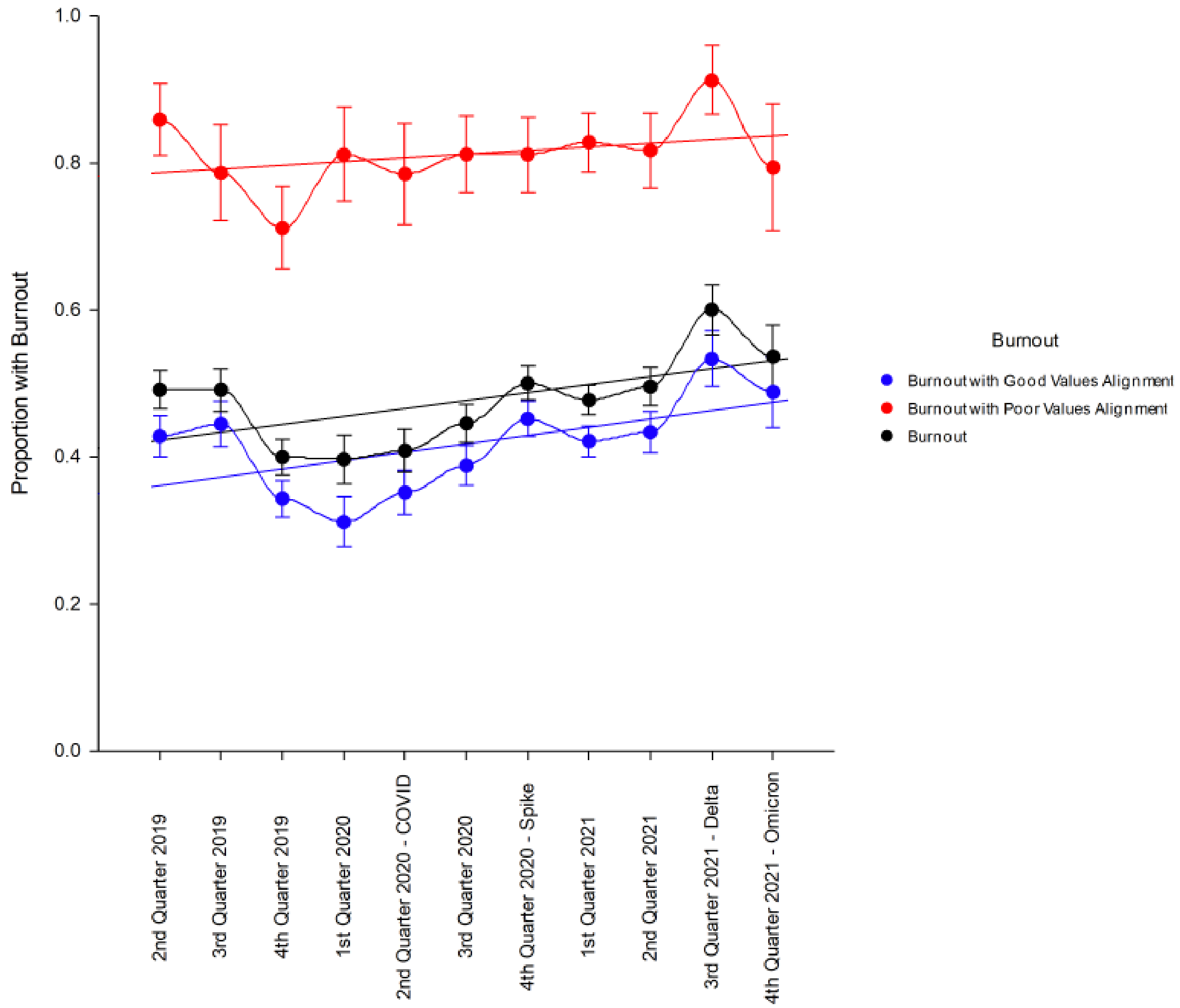

© 2022 Linzer M et al. *JAMA Health Forum.*

eFigure 3, Panel B. Trends in burnout with excessive home electronic health record (EHR) use

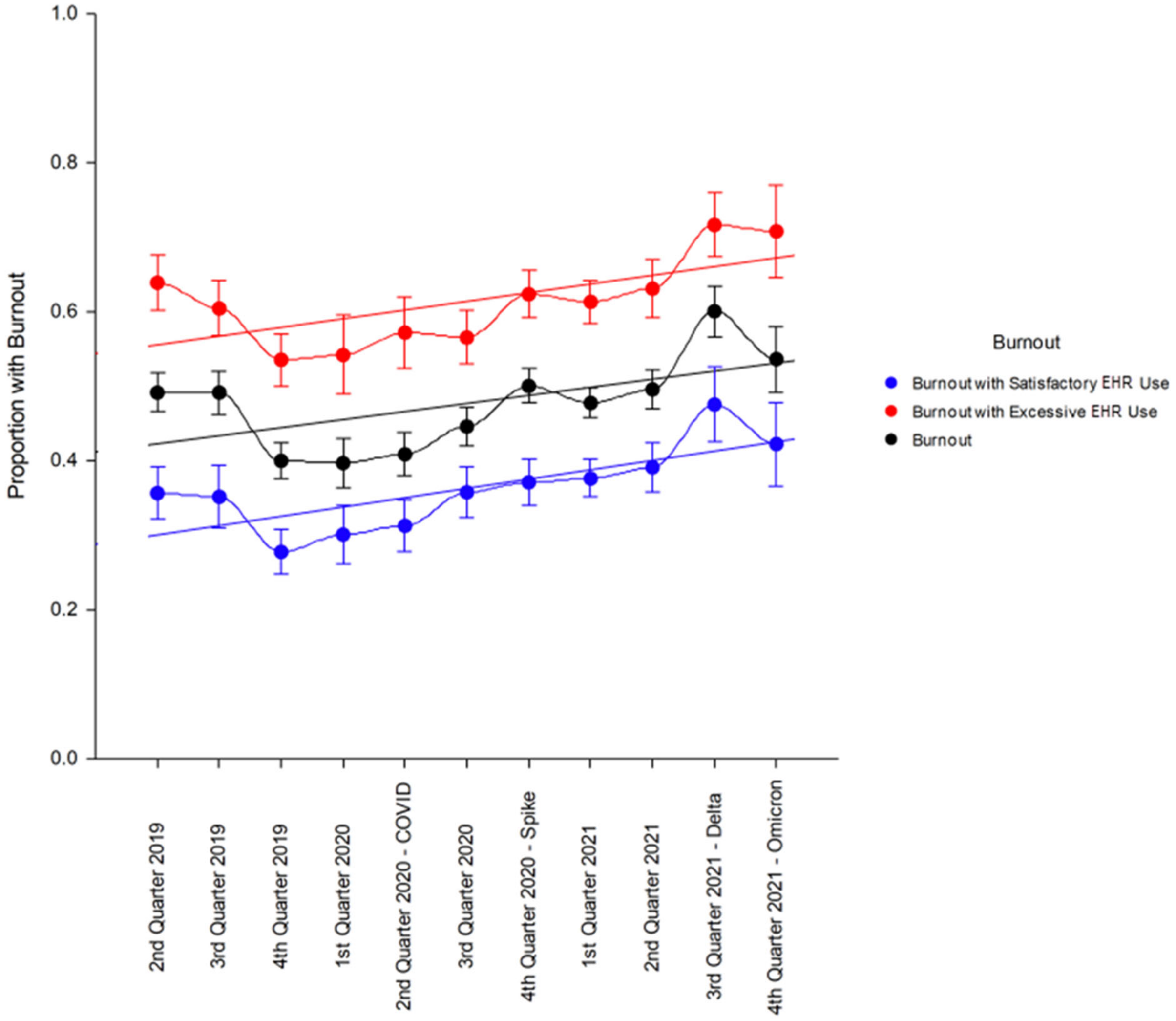

© 2022 Linzer M et al. *JAMA Health Forum.*

eFigure 4, Panel A. Trends in satisfaction with feeling valued

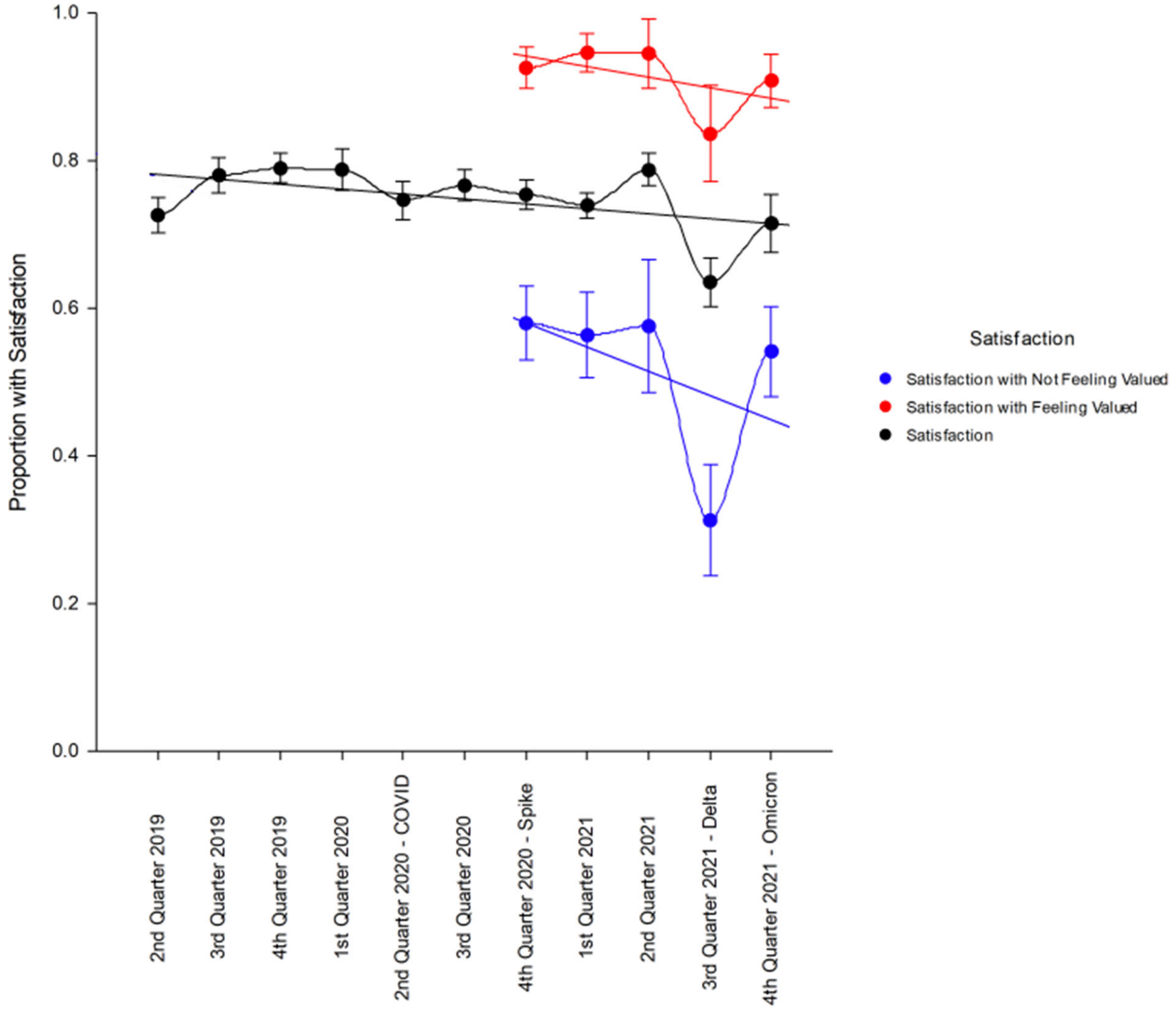

© 2022 Linzer M et al. *JAMA Health Forum.*

eFigure 4, Panel B. Changes in satisfaction with high vs low work control

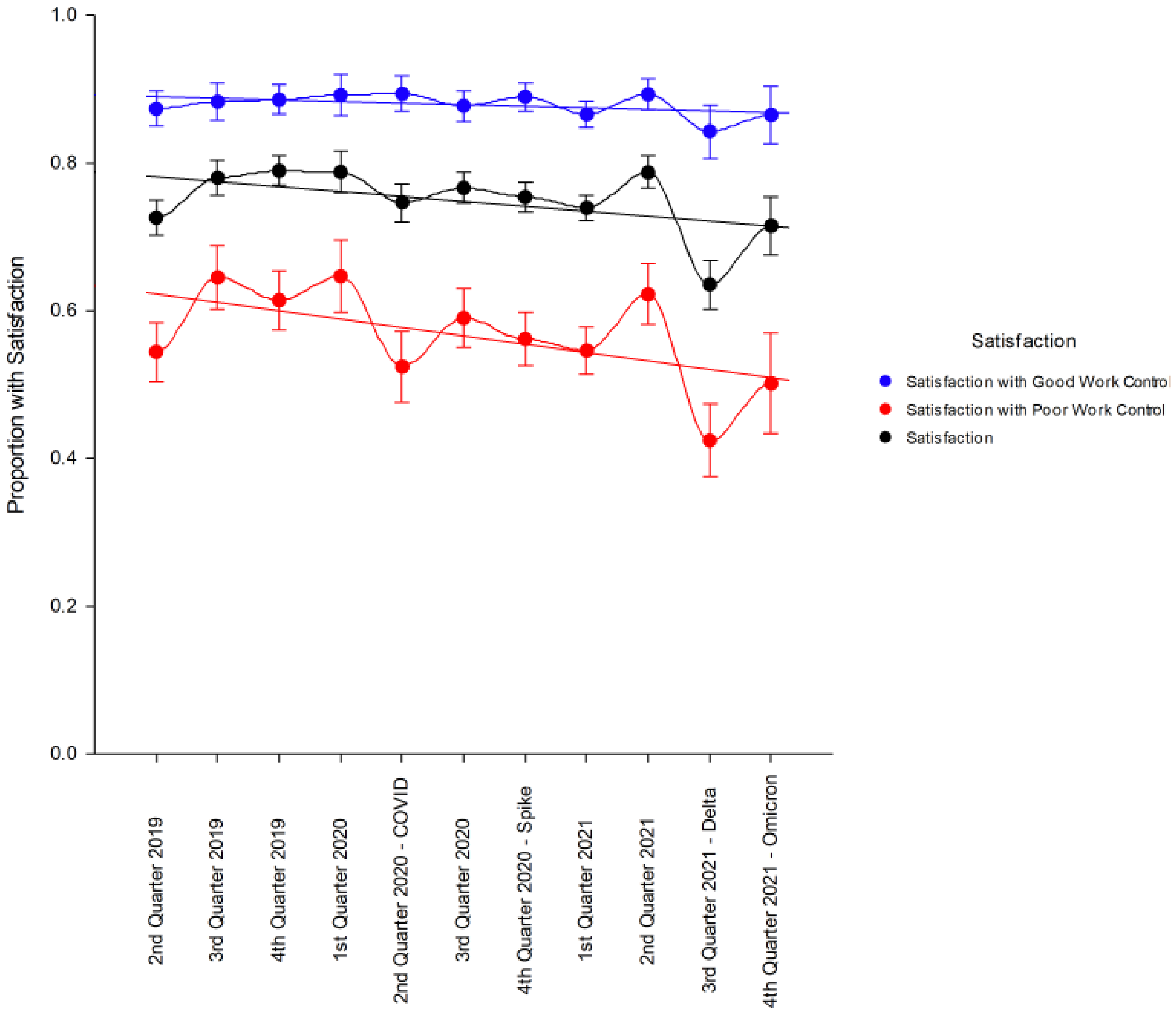

© 2022 Linzer M et al. *JAMA Health Forum.*

eFigure 4, Panel C. Changes in satisfaction with excessive home electronic health record (EHR) use

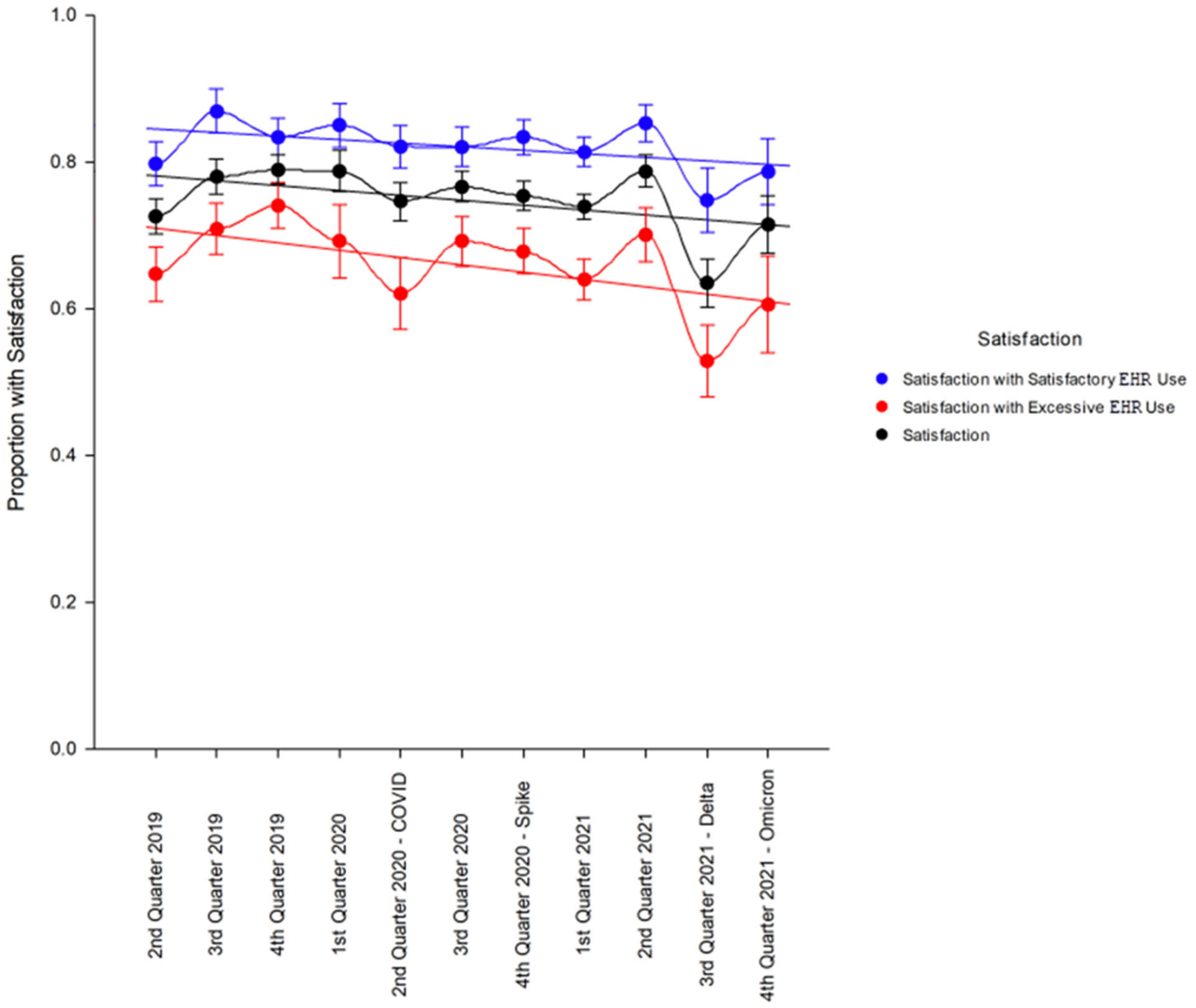

© 2022 Linzer M et al. *JAMA Health Forum.*

eFigure 5, Panel A. Trends in intent to leave with feeling valued vs not feeling valued

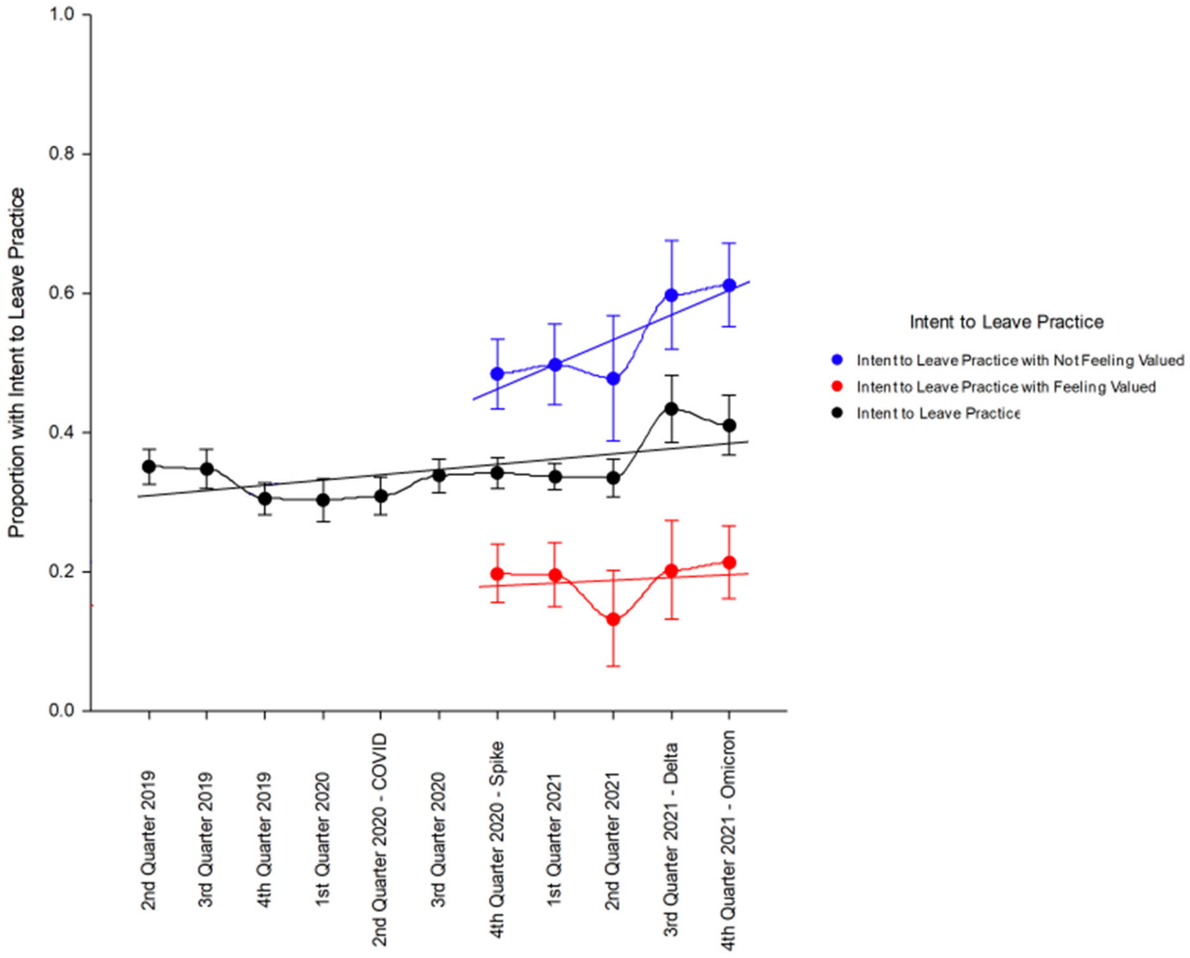

© 2022 Linzer M et al. *JAMA Health Forum.*

eFigure 5, Panel B. Changes in intent to leave with high vs low work control

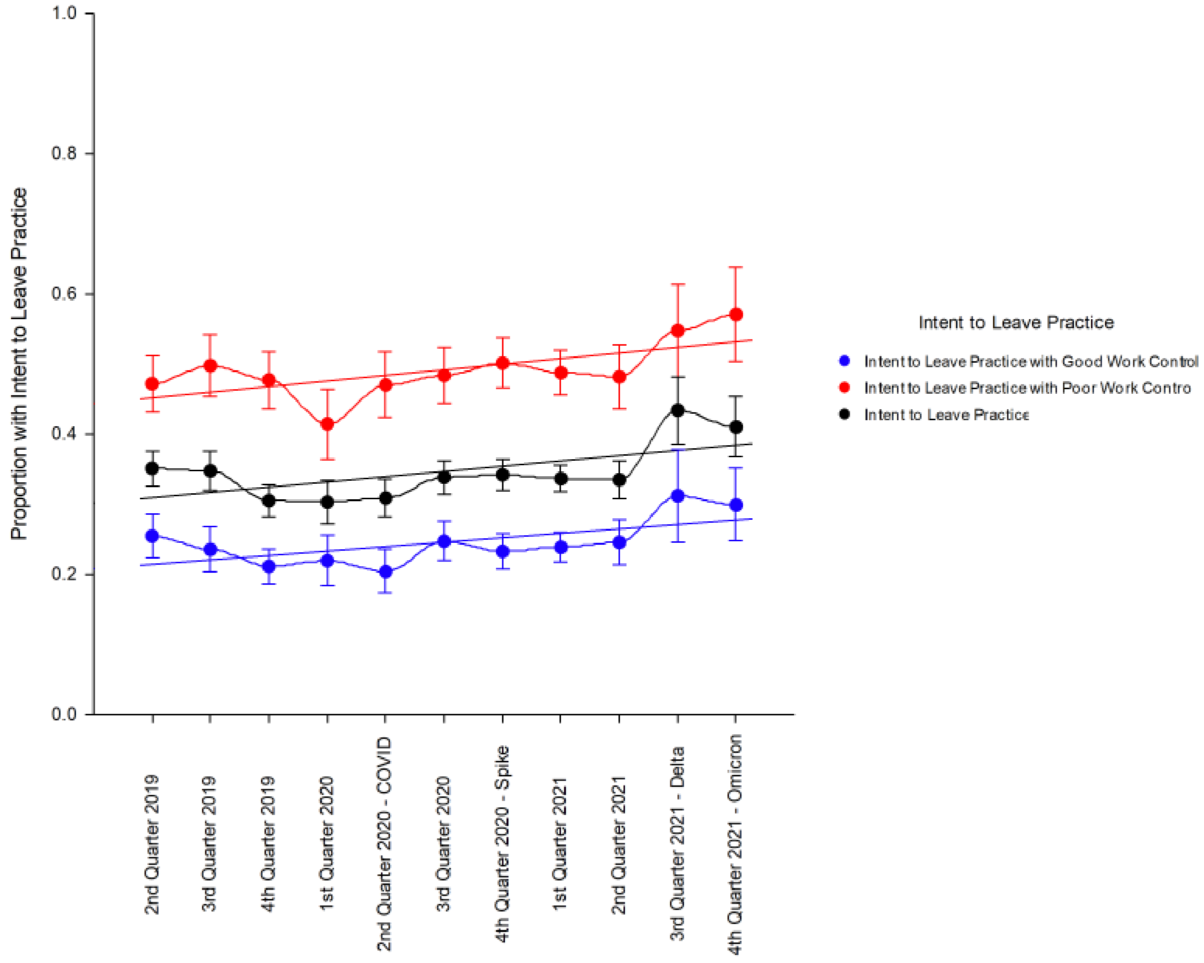

© 2022 Linzer M et al. *JAMA Health Forum.*

eFigure 5, Panel C. Changes in intent to leave with good vs poor teamwork

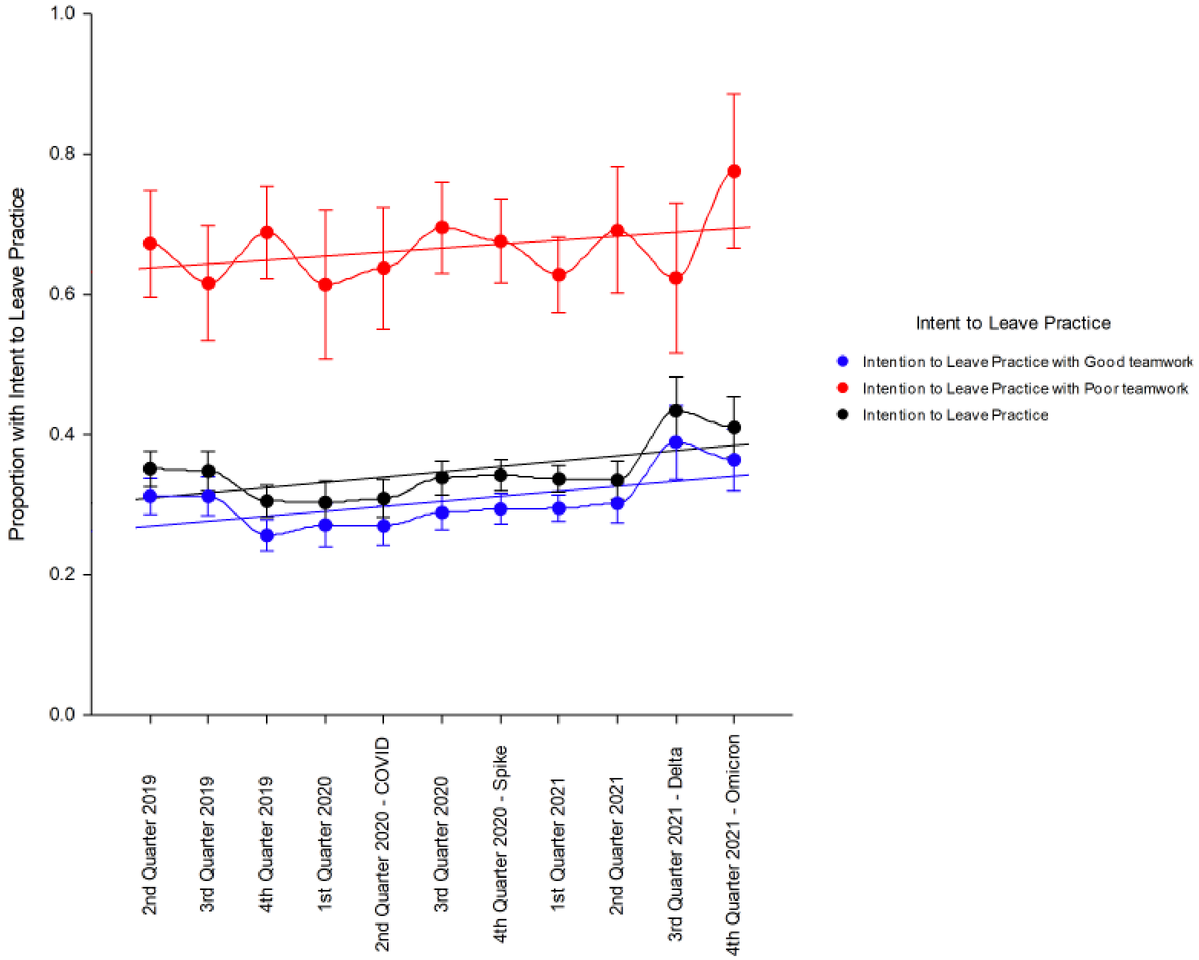

© 2022 Linzer M et al. *JAMA Health Forum.*

eFigure 6. Primary care vs subspecialty care trends in burnout 2019-21

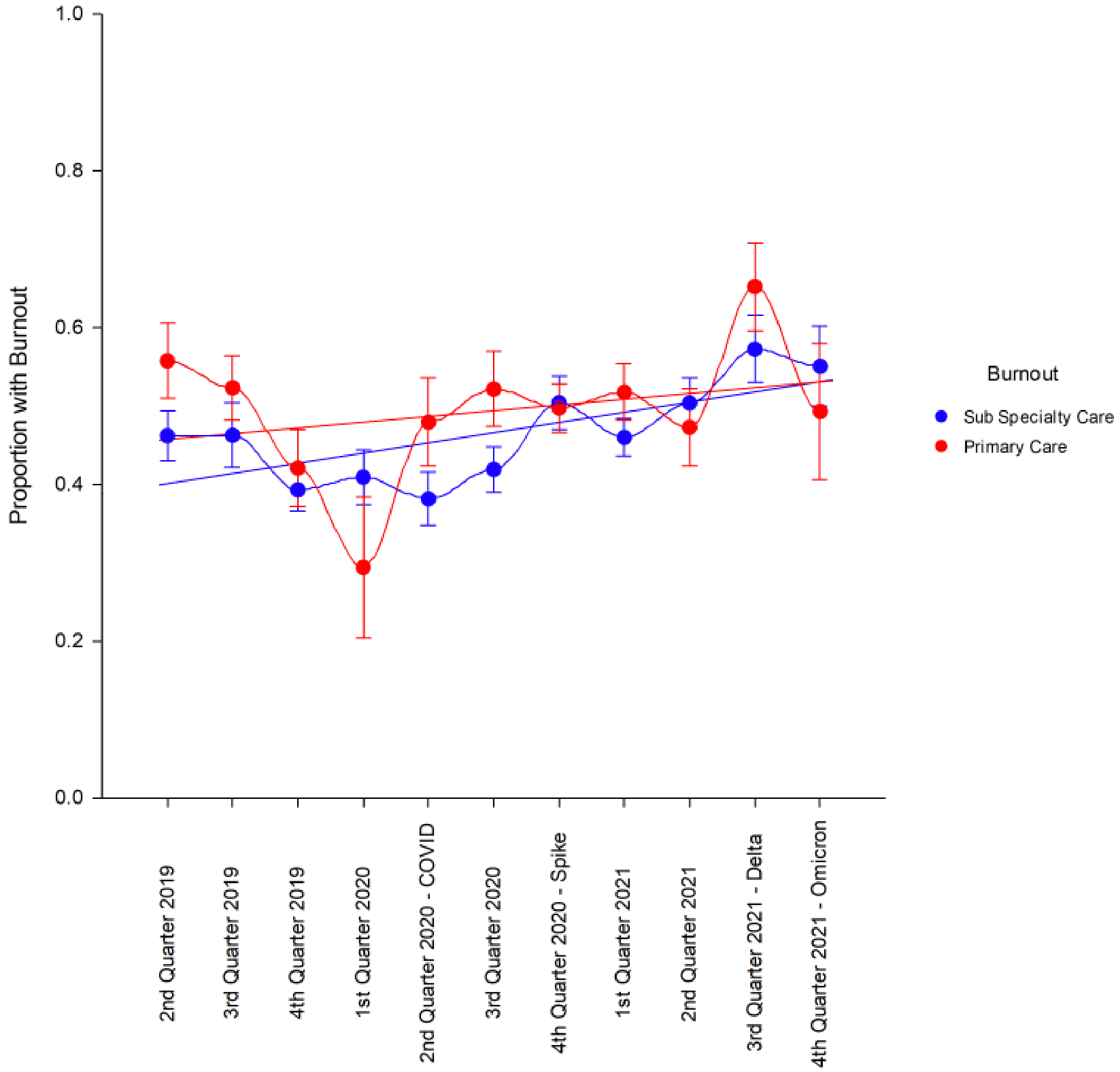

Legend. Primary care burnout rates tended to be higher than in subspecialty care, but the gap appears to be narrowing.

© 2022 Linzer M et al. *JAMA Health Forum.*
